# Integrative Phenotypic and Genomic Analysis Reveals Antimicrobial and Stress-Resistance Mechanisms of *Lacticaseibacillus rhamnosus* MG0718 as a Promising Probiotic Candidate for Food Applications

**DOI:** 10.3390/microorganisms14061290

**Published:** 2026-06-07

**Authors:** Yangyan Yin, Yanling Huang, Chunling Li, Zhe Pei, Changting Li, Zhongwei Chen, Huili Bai, Chunxia Ma, Jun Li, Hailan Chen, Hao Peng

**Affiliations:** 1 Guangxi Key Laboratory of Animal Breeding, Disease Control and Prevention, College of Animal Science and Technology, Guangxi University, Nanning 530004, China; 15177906141@163.com (Y.Y.); hyl145920@163.com (Y.H.); 18376441200@163.com (C.L.); baihuili2020@163.com (H.B.); machunxia1213@163.com (C.M.); 2 Key Laboratory of China (Guangxi)-ASEAN Cross-Border Animal Disease Prevention and Control, Ministry of Agriculture and Rural Affairs of China, Nanning 530001, China; lctyq0508@163.com (C.L.); chen_zhong-wei@163.com (Z.C.); jlee9981@163.com (J.L.); 3 Guangxi Key Laboratory of Veterinary Biotechnology, Guangxi Veterinary Research Institute, Nanning 530001, China; 4 Department of Engineering, Virginia Tech, Blacksburg, VA 24061, USA; topeizhe@hotmail.com

**Keywords:** *Lacticaseibacillus rhamnosus* MG0718, whole-genome sequencing, stress resistance genes, environmental stress tolerance, bacteriocin gene cluster, foodborne pathogen control

## Abstract

Lactobacilli species have emerged as a focal point in food microbiology due to their core probiotic properties, including the regulation of intestinal homeostasis and the enhancement of immunity. This study focuses on *Lacticaseibacillus rhamnosus* MG0718 (hereinafter referred to as MG0718), employing a combined approach of phenotypic evaluation and whole-genome sequencing to assess its probiotic potential and analyze the correlation between its phenotype and genotype. *In vitro* experiments demonstrated that MG0718 possesses broad-spectrum antibacterial activity against pathogenic bacteria. *In vitro* experiments showed that MG0718 had broad-spectrum antibacterial activity against pathogenic bacteria such as *Escherichia coli* (*E. coli*), with an inhibition zone diameter of up to 13.67 ± 1.56 mm. It survived pH 2.5 for 6 h with only a 1.72 log10 reduction, and showed 0.78 and 1.11 log10 CFU/mL reductions in artificial gastric and intestinal fluids after 2 h. DPPH scavenging was 56.7% and total reducing power was 91.1%. *In vivo*, 7-day preventive administration maintained 100% survival against *S.* Typhimurium infection and alleviated weight loss. Bacterial loads in spleen, liver, and cecum dropped from 4.5, 4.5, and 4.2 to 3.6, 1.8, and 2.5 lg CFU/g, respectively. Whole-genome sequencing analysis indicated that the complete genome of MG0718 is 2,574,565 bp in length, containing 2813 CDS. Among these genomic components, 203 stress-related protein genes elucidate its superior environmental tolerance; one bacteriocin gene cluster, one EPS gene cluster and two secondary metabolite gene clusters provide the genetic basis for its antibacterial activity. Notably, no virulence factors were detected, ensuring the safety of the strain for application. In summary, the functional phenotypes of MG0718 are highly consistent with its genetic characteristics, identifying it as a probiotic candidate of significant developmental value. Future research should focus on clinical trials to further verify its practical benefits for human intestinal health and immunomodulation, thereby providing a robust scientific basis for its application in functional foods.

## 1. Introduction

Probiotics are defined as viable microorganisms that confer health benefits to the host [1]. As microorganisms with specific functional capabilities, they have demonstrated immense potential in food preservation, quality enhancement, and the protection of consumer health. Over the past two decades, families such as *Lactobacillaceae* and *Bifidobacteriaceae*, along with certain probiotic yeasts, have become the focus of research in food microbiology. A substantial body of evidence from both in vitro and in vivo models demonstrates that these strains can enhance food safety and consumer health by optimizing the gut microbiota, accelerating the repair of intestinal epithelial injury, and modulating host immunity [2]. Among these, *Lacticaseibacillus rhamnosus* (*Lbs. rhamnosus*) [3] has emerged as one of the most commercially promising strains due to its tolerance to gastric acid and bile, as well as its ability to colonize the upper small intestine [3,4,5,6]. Its surface exopolysaccharides and pili structures significantly enhance adhesion efficiency to intestinal epithelial cells, laying the foundation for long-term colonization and sustained metabolic activity [7,8,9].

Since the completion of the first whole-genome sequence (WGS) of *Lactococcus lactis* in 2001, whole-genome sequencing has superseded traditional identification methods, becoming a core tool for elucidating the genetic background and functional potential of probiotics [10]. Hundreds of complete maps for lactobacilli strains are now available in public databases. Through pan-genome analysis, comparative genomics, and functional annotation, researchers can rapidly identify key genes associated with bacteriocin synthesis, acid and bile salt tolerance, and immunomodulation [11,12]. Currently, the integration of WGS and bioinformatics allows for a comprehensive exploration of strain genetic and biological characteristics with higher resolution and sensitivity. This approach offers valuable insights into genetic information, evolutionary relationships, physiological traits, probiotic potential, and safety profiles [13]. However, it must be acknowledged that phenotypic analysis remains crucial for the discovery of probiotics, as sequencing processes may contain errors, and the consistency between genomic sequences and existing databases is finite [14].

For example, *Lbs. rhamnosus* KF7 isolated from kefir was evaluated through whole-genome sequencing, phenotypic safety assays, and a *Caenorhabditis elegans* in vivo model. This confirmed the absence of virulence factors and antibiotic resistance genes and demonstrated colonization potential [15]. Similarly, strain *Lbs. rhamnosus* SAL2 was characterized through hybrid Illumina/PacBio sequencing and multidimensional phenotypic benchmarking against the reference strain *Lbs. rhamnosus* GG. The results revealed comparable safety and superior antioxidant performance [16]. Other studies, such as those on dairy-derived *Lbs. rhamnosus* 044AE [17], also combined in vitro probiotic assays with genomic annotation to identify stress-response genes and bacteriocin clusters. Together, these studies show that phenotype-genotype integration is a powerful tool for probiotic screening.

However, several key gaps remain. Most screening studies are still limited to in vitro assays. They lack in vivo pathogen challenge validation to confirm protective efficacy under real host infection conditions. Although phenotypic and genomic data are often presented together, the mechanistic link between specific genetic loci and observed functional advantages is frequently unclear. This limits the ability to predict phenotype from genotype. Moreover, probiotic traits are highly strain-specific. The functional mechanisms of newly isolated strains require independent characterization, rather than inference from model strains such as *Lbs. rhamnosus* GG.

Notably, recent studies using *Salmonella enterica* serovar Typhimurium (*S.* Typhimurium) murine infection models have shown that in vivo validation is essential to confirm probiotic protective effects. This was demonstrated by *Lbs. rhamnosus* P118 [18], which significantly improved survival rates and reduced pathogen burden in infected mice through microbe-derived indole metabolites [15,16].

To address this, the present study focuses on *Lbs. rhamnosus* MG0718 (hereinafter referred to as MG0718), a strain isolated from Sour Bamboo Shoot that exhibits favorable antibacterial activity, hereinafter designated as MG0718. We first systematically determined key probiotic phenotypes, including acid and bile salt tolerance, cell adhesion, and antimicrobial spectrum. Subsequently, we utilized Illumina NovaSeq to complete whole-genome sequencing, mining for potential bacteriocin and stress tolerance genes through comparative genomics and functional annotation. On this basis, we established a mouse model of *S. *Typhimurium infection to evaluate the protective effects of MG0718 on intestinal colonization, pathological injury, inflammatory cytokine levels, and survival rates. By correlating the genome, phenotype, and in vivo protective efficacy, we aim to provide theoretical and experimental evidence for the development of *Lbs. rhamnosus* preparations that function as both preservatives and intestinal protectants.

## 2. Materials and Methods

### 2.1. Strains and Culture Methods

*S.* Typhimurium SM022 was generously provided by Professor Alejandro Aballay of Duke University, USA. *Escherichia coli* ATCC 25922, *Staphylococcus aureus* subsp. *aureus* ATCC 6583 (*S. aureus* ATCC 6583), and MG0718 (isolated from traditional Sour Bamboo Shoot in the Guangxi region) were obtained from the Guangxi Veterinary Research Institute (Nanning, China). MG0718 was inoculated into MRS medium (Haibo, Shanghai, China) and incubated at 37 °C for 18 h. Cell-free supernatant (CFS) was collected after centrifugation (8000× *g*, 4 °C, 10 min). To obtain bacterial suspensions (BS) (Haibo, Shanghai), strains’ cells were washed three times with phosphate-buffered saline (PBS) and adjusted to 1 × 10^9^ CFU/mL. Pathogenic strains’cells were inoculated into LB medium (Haibo, Shanghai), incubated at 37 °C for 18 h, centrifuged (8000× *g*, 4 °C, 10 min), washed three times with PBS, and adjusted to 1 × 10^9^ CFU/mL.

### 2.2. In Vitro Antibacterial Experiment of MG0718

#### 2.2.1. The Oxford Cup Assay

Modified from He et al. [19], the pathogenic strains were used as indicator bacteria to observe the inhibitory capability against common pathogens. Pathogenic bacteria were cultured to the logarithmic growth phase. A 100 μL aliquot of a bacterial suspension containing 1.0 × 10^6^–10^7^ CFU/mL was spread onto an LB agar plate. Oxford cups were placed on LB plates spread with pathogenic bacteria BS. Subsequently, 200 µL of the test bacterial solution or supernatant was added to the Oxford cups and incubated at 37 °C for 18 h. The diameter of the inhibition zone was observed, with three replicates set for each strain.

#### 2.2.2. Characterization of MG0718 Antimicrobial Substances

Following the method of Scillato M et al. [20]. The CFS was treated as follows:

(1) pH Neutralization: The pH of the CFS was adjusted to 6.5 using 1 mol/L sterile NaOH. MRS medium adjusted to pH 6.5 with lactic acid and acetic acid served as blank controls.
(2) The CFS was adjusted to pH 6.5, and catalase (final concentration 5 mg/L) was added, followed by a water bath at 37 °C for 2 h. MRS medium adjusted to pH 6.5 with catalase added served as a blank control.
(3) Proteolytic Enzyme Treatment: To determine the proteinaceous nature of the antimicrobial substances, trypsin, papain, proteinase K (Haibo, Shanghai), and pepsin (final concentration 1 mg/mL) were added to the CFS separately and incubated in a water bath at 37 °C for 2 h.
(4) Untreated fermentation supernatant served as the control. 200 µL of the treated CFS was added to Oxford cups, incubated at 37 °C for 18 h, and the diameter of the inhibition zone was measured in triplicate.

### 2.3. Prophylactic Protection of MG0718 Against S. Typhimurium Infection in C57BL/6 Mice

#### 2.3.1. Strain Preparation

*S. *Typhimurium SM022 and MG0718 were grown to the logarithmic phase in LB broth and MRS broth (Haibo, Shanghai), respectively. Bacterial cells were collected by centrifugation at 894× *g* for 10 min, washed twice with PBS, and adjusted to a concentration of 1 × 10^8^ CFU/mL prior to gavage.

#### 2.3.2. Experimental Grouping

Thirty-two 4-week-old female C57BL/6 mice were housed under standardized conditions (22–26 °C, 20% humidity, 12 h light/dark cycle) with ad libitum access to standard diet and water. After one week of acclimatization, mice were randomly divided into four groups (*n* = 8 per group). All animal experiments described in this paper were conducted in strict accordance with the National Standard of the People’s Republic of China, Guidelines for the Ethical Review of Laboratory Animal Welfare (GB/T 35892-2018), and the Guidance on the Humane Treatment of Laboratory Animals. The experimental protocol was reviewed and approved by the Laboratory Animal Ethics Committee of the Guangxi Veterinary Research Institute (Approval No.: GXV-2026-005). Throughout the experiments, all animals were treated humanely and in accordance with the ‘3Rs’ principle, minimising both animal suffering and the number of animals used.

**Figure 1:**
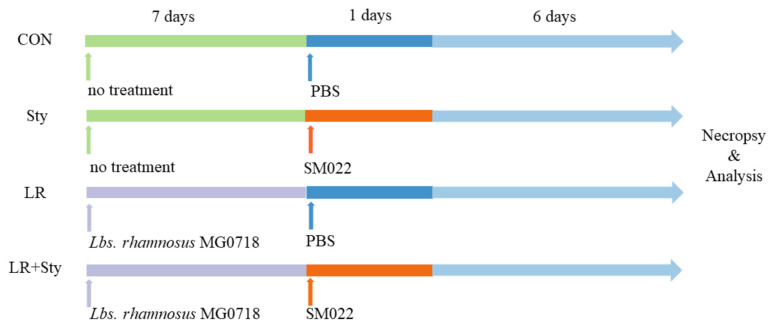
Prophylactic protection of MG0718 against SM022 infection in C57BL/6 mice.

Negative Control (CON): No treatment; MG0718 Control (LR): Received MG0718;

MG0718 Prevention (LR + Sty): Received MG0718 followed by *S. *Typhimurium SM022; Positive Control (Sty): Received *S. *Typhimurium SM022; Detailed grouping is shown in Figure 1. Mice were euthanized and analyzed 14 days after *S. *Typhimurium SM022 gavage.

#### 2.3.3. Detection of Body Weight Changes and Survival Rate

Mouse weight and quantity were recorded before feeding with *S. *Typhimurium SM022. Following infection, the number of surviving mice was recorded daily for 7 days to generate a survival curve. Mice were weighed prior to euthanasia to plot body weight changes.

#### 2.3.4. Detection of *S.* Typhimurium Load in Colon, Liver, and Spleen

Adapted from the article by Ou et al. [21]. The liver, spleen, and cecum of each mouse were weighed, thoroughly ground in PBS, and serially diluted (10^0^ to 10^−5^). 100 µL of each dilution was spread evenly on bismuth sulfite agar plates using sterile glass beads. After incubation at 37 °C for 18 h, Salmonella load was calculated based on colony counts in each tissue.

#### 2.3.5. Detection of Liver Lesions

Adapted from the article by Yin et al. [22]. On the 5th day challenge, three mice from each group were randomly selected for euthanasia. Livers were aseptically removed and immediately fixed in 4% formaldehyde solution for 48 h. Fixed tissue samples were transported to the Guangxi University of Chinese Medicine for histological processing and analysis.

#### 2.3.6. Measurement of Inflammatory Factors

Adapted from the article by Li et al. [23]. Total RNA was extracted from liver tissue using the Cwbio kit (CW0581S) (Shanghai, Shenggong, China). cDNA synthesis was performed using the Cwbio kit (CW2020M) (Shanghai Shenggong). Quantitative analysis was conducted using Sangon Biotech SYBR Green qPCR reagent (B690016-0005) (Shanghai Shenggong). Gene expression differences between groups were calculated using the 2^−ΔΔCt^ method, normalized to *β*-actin.

### 2.4. Biological Characteristics of MG0718 in In Vitro Culture

#### 2.4.1. Acid Resistance Test

Adapted from the article by Jankoski et al. [24]. PBS (1×) was adjusted to pH 2, 2.5, 3, and 3.5 using 0.1 mol/L HCl and sterilized at 121 °C for 20 min. Bacterial samples were resuspended in an equal volume of PBS and inoculated at 10% into the pH-adjusted PBS. Cultures were incubated at 37 °C. Samples were taken every 2 h, serially diluted with phosphate-buffered saline, and plated to count viable colonies from 0 to 6 h.

#### 2.4.2. Bile Salt Tolerance Test

Adapted from the article by Jankoski et al. [24]. Bacteria were inoculated at 10% into solutions containing 0.03%, 0.1%, 0.2%, and 0.3% bile salts (Haibo, Shanghai). Cultures were incubated at 37 °C. Samples were collected hourly, diluted in PBS, and plated to determine viable counts over 3 h.

#### 2.4.3. Artificial Gastrointestinal Fluid Tolerance Test

Adapted from the article by Jankoski et al. [24]. Bacteria were inoculated at 10% into simulated intestinal or gastric fluid. Samples were taken at 0, 30, 60, 90, and 120 min for colony counting. Experiments were performed in triplicate.

#### 2.4.4. Temperature Tolerance Test

Adapted from the article by Reuben et al. [25]. Bacterial suspensions were inoculated at 10% (*v*/*v*) into 5 mL PBS. For high-temperature tolerance, samples were incubated at 37, 40, 60, 70, 80, and 90 °C water baths for 30 min. For low-temperature tolerance, samples were stored at −20, 0, 5, and 10 °C for 48 h, with room temperature as a control. Viable counts were determined by plate counting in triplicate.

### 2.5. Bacterial Adhesion Ability Detection

#### 2.5.1. Auto-Aggregation Detection

Adapted from the article by Chaudhari et al. [26]. After 18 h of culture, activated bacteria were centrifuged (8000× *g*, 5 min), washed twice with PBS, and resuspended. The initial absorbance (OD_600_ recorded as D_0_) was measured. The suspension was vortexed for 30 s and incubated statically at 30 °C. Subsequent absorbance values (D_t_) were recorded at 1, 2, 3, 4, and 24 h. The auto-aggregation rate (A, %) was calculated as:

$$A=\left(1-\frac{D_{t}}{D_{0}}\right)\times 100\%$$

#### 2.5.2. Hydrophobicity Determination

Adapted from the article by Apostolakos et al. [27]. 2 mL of bacterial suspension was mixed with an equal volume of hexadecane, vortexed for 1 min, and allowed to separate at room temperature for 30 min. The OD_600_ of the aqueous phase (D_1_) was measured. Hydrophobicity (H, %) was calculated as:

$$H=\left(1-\frac{D_{1}}{D_{0}}\right)\times 100\%$$

#### 2.5.3. Determination of Antioxidant Activity

##### DPPH Radical Scavenging Activity

Adapted from the article by Yin et al. [28]. 1 mL of CFS was mixed with 2 mL of DPPH (Solarbio, Beijing, China) ethanol solution (0.2 mmol/L), vortexed, and incubated in the dark at room temperature for 30 min. After centrifugation (3578× *g*, 10 min), absorbance was measured at 517 nm. Scavenging capacity was calculated as:

$$\mathrm{DPPH}\;\mathrm{scavenging}\;(\%)=(1-\frac{A_{1}-A_{2}}{A_{0}})\times 100$$

A_0_: Absorbance value measured by the same treatment with an equal volume of anhydrous ethanol instead of the sample to be tested.
A_1_: absorbance value of the experimental group.
A_2_: equal volume of anhydrous ethanol instead of DPPH solution; the absorbance value measured by the same treatment.

##### Superoxide Anion Radical Scavenging Activity

Adapted from the article by Yin et al. [28]. 3.4 mL of Tris-HCl (50 mmol/L, pH 8.2) (Solarbio, Beijing, China) was mixed with 0.5 mL pyrogallol (50 mmol/L) (Solarbio, Beijing) and 1 mL of sample, then incubated at 25 °C for 4 min. The reaction was terminated with 0.1 mL of 8 mol/L HCl, and absorbance was measured at 325 nm. Scavenging rate was calculated as:

$${O_{2}}^{-}\;\mathrm{scavenging}\;\mathrm{rate}\;(\%)=\frac{A_{3}-A_{2}}{A_{1}-A_{0}}\times 100$$

A_0_: Equal volumes of water were used to replace the samples to be tested and o-triacontanol, respectively, and the absorbance values were measured by the same treatment.
A_1_: equal volume of water instead of the sample to be tested, the same treatment of the measured absorbance values.
A_2_: equal volume of water instead of o-o-benzenetriol, the same treatment of the measured absorbance values.
A_3_: absorbance value of the experimental group.

##### Determination of Total Reducing Power (TP)

Adapted from the article by Yin et al. [28]. A mixture of 0.5 mL potassium permanganate (1%) (Solarbio, Beijing), 0.5 mL PBS, and 0.5 mL sample was incubated at 50 °C for 20 min. Then, 0.5 mL trichloroacetic acid (10%) was added, followed by centrifugation (224× *g*, 10 min). The supernatant was mixed with an equal volume of FeCl_3_ (0.1%), and absorbance was measured at 700 nm. TP was calculated as:

$$\mathrm{TP}\;(\%)=\frac{A_{1}-A_{0}}{A_{1}}\times 100$$

A_0_: Absorbance value measured by the same treatment with PBS instead of the sample to be tested.
A_1_: absorbance value of the experimental group.

### 2.6. Genomic DNA Extraction, Library Construction, and Sequencing

Genomic DNA was extracted and purified using the Qiagen DNA extraction kit (Qiagen, Düsseldorf, Germany) following the manufacturer’s instructions. Sequencing library construction and paired-end sequencing were performed by OE Biotech Co., Ltd. (Shanghai, China). Briefly, 2.5 μg of genomic DNA was fragmented, end-repaired, and A-tailed for library preparation. Paired-end sequencing (2 × 250 bp) was subsequently conducted on the Illumina platform. Raw sequencing data were quality-controlled using FastQC and assembled using Unicycler.

### 2.7. Virulence Factor Analysis

Protein coding sequences were BLASTed against the Virulence Factor Database (VFDB) with thresholds of E-value 1 × 10^−5^, identity > 60%, coverage > 70%, and gap length < 10% of total length.

### 2.8. Genome Annotation

The BLAST software (BLAST 2.14.0) was used to perform sequence alignments of the genes against the eggNOG/COG (http://www.ncbi.nlm.nih.gov/COG/, accessed on 18 August 2025), CAZy (http://www.cazy.org/ accessed on 18 August 2025), and KEGG (http://www.genome.jp/kegg/ accessed on 18 August 2025) databases to obtain gene annotation information. Specifically, the annotation of genes involved in carbohydrate metabolism pathways in the *Lbs. rhamnosus* MG0718 strain was primarily analyzed using the KEGG (Kyoto Encyclopedia of Genes and Genomes) and COG (Clusters of Orthologous Groups) pathway databases, while the functional genes of carbohydrate-active enzymes were analyzed using the CAZy (Carbohydrate-Active Enzymes) database.

### 2.9. Statistical Analysis

All experiments were performed in triplicate. Data were analyzed using Excel and open-source statistical software, with one-way analysis of variance (ANOVA) employed for multiple comparisons. Graphs were generated using QtiPlot. All data are expressed as mean ± SEM, * *p* < 0.5, ** *p* < 0.01, *** *p* < 0.001, **** *p* < 0.0001, when compared with the Model group.

## 3. Results

### 3.1. Bacteriostatic Phenotype

#### 3.1.1. Results of Oxford Cup Experiments, Acid-Exclusion, H-Exclusion and Protein-Exclusion Tests

As shown in Table 1 and Figure S1, the CFS of MG0718 inhibited all three foodborne pathogens, with the strongest effect observed against *E. coli* ATCC 25922 (13.67 ± 1.56 mm).

Table 1, Table 2 and Table 3 (see also Figure S2) indicate that upon the exclusion of acid, hydrogen peroxide, and proteins, the inhibitory effect of MG0718 CFS on pathogens was largely negated when pH was neutralized. This suggests that inhibition is primarily mediated by acid production. The exclusion of pepsin appeared effective (retaining inhibition) likely because the low pH required for pepsin activation allowed the acidic inhibition mechanism to persist.

#### 3.1.2. In Vivo Experiment of MG0718 Preventing *S.* Typhimurium Infection

**Figure 2:**
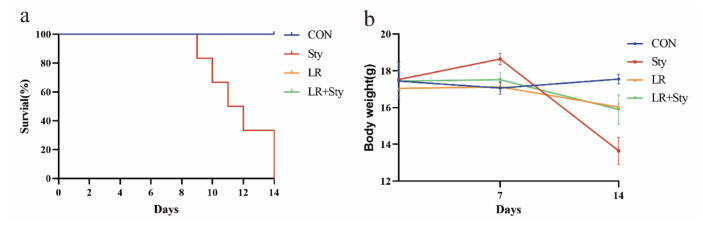
Prophylactic protection of MG0718 against *S. *Typhimurium infection in C57BL/6 mice: (**a**): Survival rates in different groups; (**b**): Changes in body weight in different groups, in different groups. *n* = 8 per group.

Prophylactic feeding of MG0718 significantly improved survival rates in *S. *Typhimurium-infected mice (Figure 2a). The survival rate for LR, CON, and LR + Sty groups was 100%, whereas the Sty group was 0%. Additionally, MG0718 treatment mitigated infection-induced weight loss (Figure 2b), where the SM022 group showed significant weight reduction while the LR-treated group showed alleviated weight loss.

#### 3.1.3. MG0718 Reduces Infection of the Liver, Spleen and Caecum by *S.* Typhimurium in Mice

**Figure 3:**
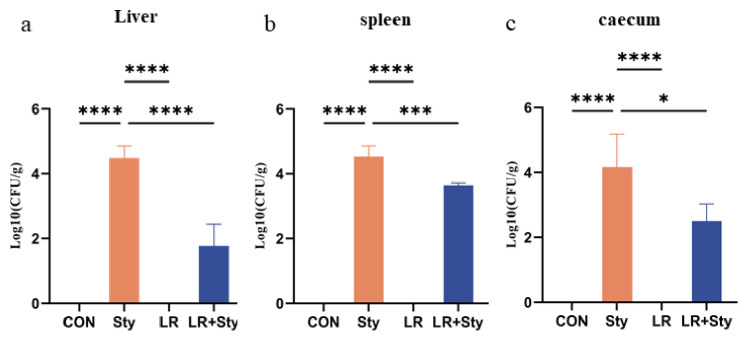
MG0718 alleviates *S. *Typhimurium infestation. Effect of MG0718 on Bacterial Load in Various Tissues of Mice Infected with *S. *Typhimurium; (**a**): Liver; (**b**): spleen; (**c**): caecum. The levels of significance are defined as follows: *, *p* < 0.05; ***, *p* < 0.001; ****, *p* < 0.0001.

*S. *Typhimurium relies on intestinal colonization to induce disease [29]. Following infection, bacterial loads in the spleen, liver, and cecum of the Sty group increased significantly to 4.5, 4.5, and 4.2 lg CFU/g (Figure 3), respectively. In contrast, the LR + Sty group showed significantly lower loads (3.6, 1.8, and 2.5 lg CFU/g, respectively), indicating that MG0718 effectively reduced pathogen burden.

#### 3.1.4. Mitigation of Liver Pathology

**Figure 4:**
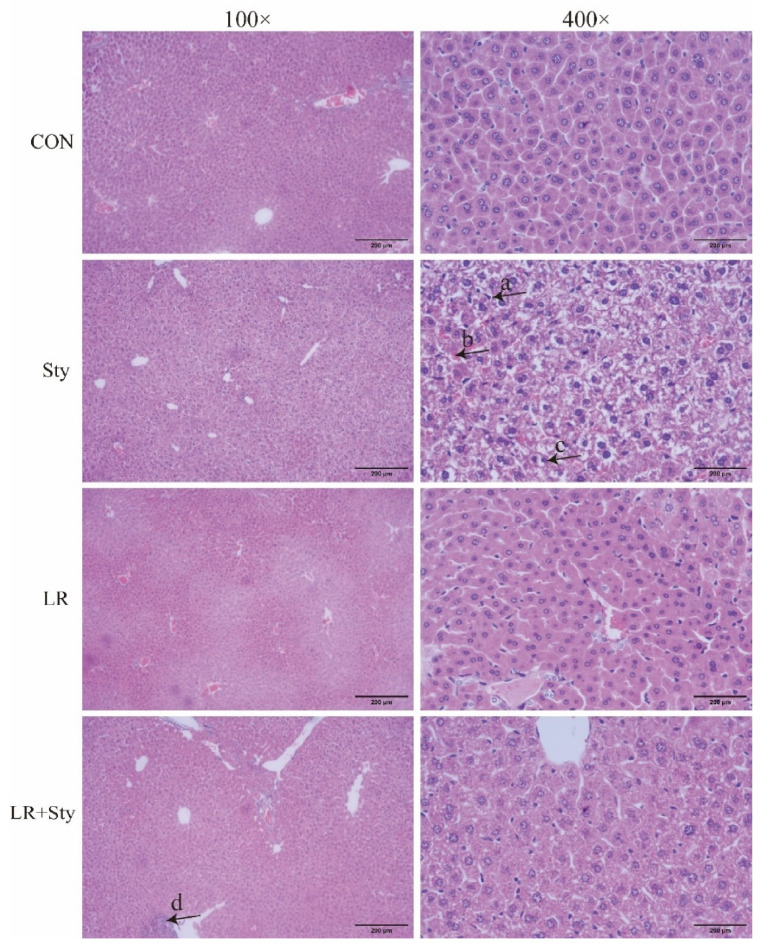
Annotations on Mouse Liver Sections: shows the mouse liver’s HE staining results. (Arrow a) nuclear condensation; (Arrow b) mild bleeding; (Arrow c) hepatocyte vacuolar degradation; and (Arrow d) mild infiltration of inflammatory cells. Control group: On days 8 and 9, mice were given PBS orally. LR group: Mice were given PBS on days 8 and 9 and MG0718 orally from day 1 to day 7. LR + Sty group: Oral MG0718 was given to the mice from day 1 to day 7, and *S. *Typhimurium was given on days 8 and 9. Sty group: On days 8 and 9, mice were given *S. *Typhimurium.

Histopathological analysis (Figure 4) showed that the Sty group exhibited blurred tissue structure, cellular swelling, nuclear condensation (pyknosis), hepatocyte vacuolar degeneration, and local hemorrhage. Conversely, the LR + Sty group displayed intact tissue structure, organized hepatocyte arrangement, and limited inflammatory infiltration, demonstrating that MG0718 alleviated liver damage.

#### 3.1.5. Alleviation of Intestinal Inflammation

**Figure 5:**
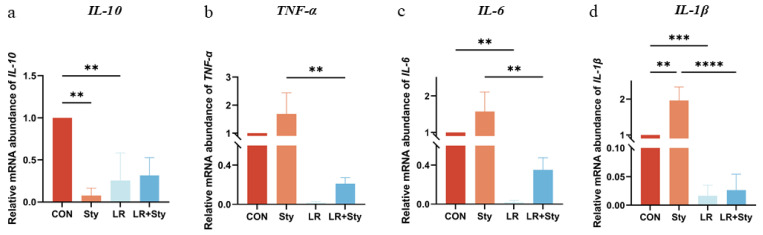
MG0718 ameliorates intestinal injury in mice caused by *S. *Typhimurium. mRNA relative expression of *IL-10* (**a**), *TNF-α* (**b**), *IL-6* (**c**) and *IL-1β* (**d**) in colon tissue (*n* = 3 per group). The levels of significance are defined as follows: **, *p* < 0.01; ***, *p* < 0.001; ****, *p* < 0.0001.

As shown in Figure 5, mRNA expression levels of pro-inflammatory cytokines (*IL-6*, *TNF-α*, *IL-1β*) in the jejunum were significantly reduced in the LR + Sty group compared to the Sty group (*p* < 0.001). While *IL-10* levels decreased in the Sty group, MG0718 treatment modulated the inflammatory response. Overall, these results suggest that MG0718 may mitigate the inflammatory response caused by *Salmonella* infection.

### 3.2. Anti-Stress Phenotype

**Figure 6:**
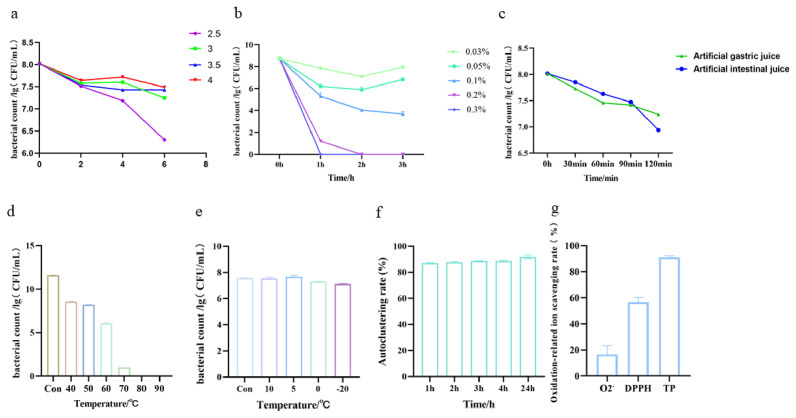
In vitro bioactivity assay results of MG0718. (**a**): Effect of different pH values on the growth of MG0718 strain at 37 °C; (**b**): Effect of different concentrations of bile salts on the growth of MG0718 strain at 37 °C; (**c**): Effect of artificial gastrointestinal fluid on the growth of MG0718 strain at 37 °C; (**d**): Effect of different high temperatures on the growth of MG0718 strain at 48 h; (**e**): Effect of different low temperatures on the growth of MG0718 strain at 48 h; (**f**): Auto-aggregation of MG0718 strain at different times at 30 °C; (**g**): Determination of antioxidant activity of MG0718 strain.

MG0718 exhibited a general decline in viability under simulated gastrointestinal and stress conditions. However, it demonstrated strong acid resistance, with only a 1.72 log10 reduction after 6 h at pH 2.5 (Figure 6a). Tolerance to artificial gastric and intestinal fluids was also robust, with reductions of only 0.78 and 1.11 log10 CFU/mL, respectively, after 2 h (Figure 6c). While sensitive to high bile salt concentrations (no survival at 0.2% after 2 h, Figure 6b), the strain showed strong cold tolerance (stable at −20 to 10 °C, Figure 6e). Thermal tolerance was moderate, with survival maintained at 60 °C (6 log10 CFU/mL) but declining at higher temperatures (Figure 6d). The auto-aggregation rate was high (>87%), while hydrophobicity was low (16.7%) (Figure 6f). Antioxidant assays revealed a DPPH scavenging capacity of 56.7% and a total reducing power of 91.1%, highlighting its potential to mitigate oxidative damage (Figure 6g).

### 3.3. Basic Features of MG0718 Genome

**Figure 7:**
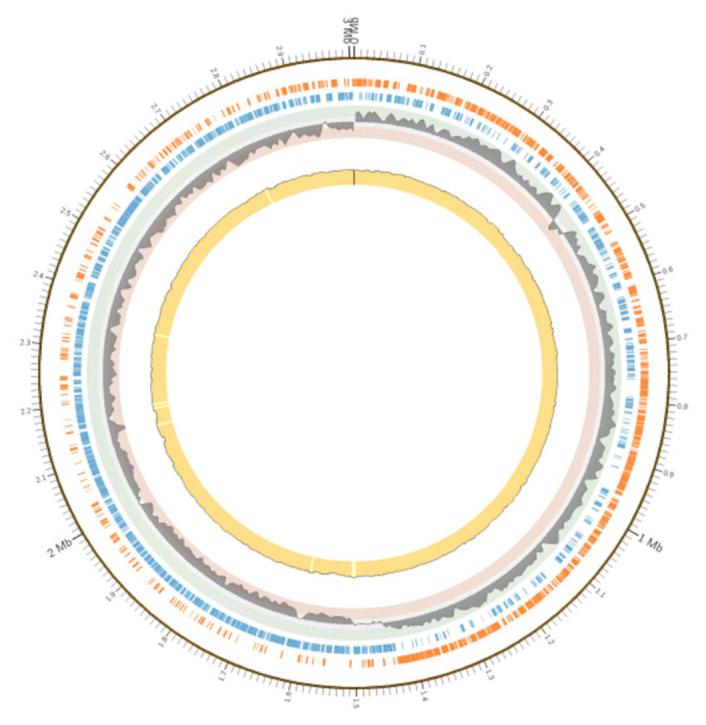
Circular map of the complete MG0718 genome. From the outside to the inside, the circles represent the following:  First circle: Genome sequence coordinates (in kb). Second circle: Coding sequences (CDS) and non-coding RNA regions (rRNA, tRNA) of the reference genome, shown in two layers. The outer layer represents the forward strand, and the inner layer represents the reverse strand. Third circle: Independent marker layer for non-coding RNA regions (rRNA, tRNA), showing the distribution of ribosomal RNA and transfer RNA across the genome. Fourth circle: GC skew curve of the genome sequence, calculated using a 2000 bp sliding window. The dashed line indicates the baseline where GC skew equals zero. Fifth circle: GC content curve of the genome sequence, calculated using a 2000 bp sliding window. The dashed line indicates the average GC content of the reference genome.

The MG0718 genome consists of one circular chromosome and four circular plasmids (Figure 7). The chromosome spans 2,574,565 bp with an average GC content of 47%. It contains 2813 predicted coding sequences (CDS) and 59 tRNA genes (Table 4).

#### 3.3.1. Functional Annotation of MG0718

**Figure 8:**
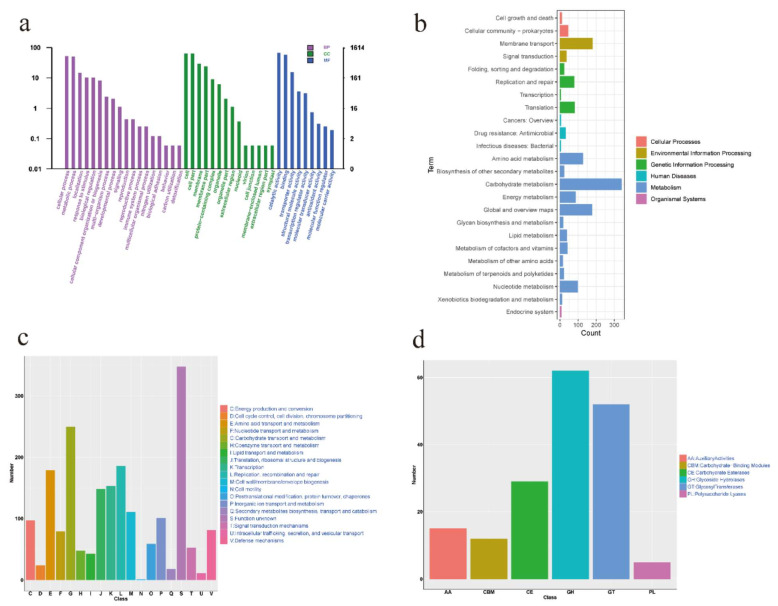
Functional annotation of the MG0718 genome. (**a**): MG0718 GO database annotation results (**b**): MG0718 KEGG database annotation results. (**c**): The number of matched genes assigned in COGs in MG0718. (**d**): MG0718 CAZy database.

GO annotation (Figure 8a) identified 8132 genes, with catalytic activity and metabolic processes being dominant. KEGG analysis (Figure 8b) annotated 1436 genes, with carbohydrate metabolism being the most enriched pathway (340 genes). COG annotation (Figure 8c) also highlighted carbohydrate transport and metabolism (250 genes). Furthermore, 111 genes were related to cell wall/membrane biogenesis, supporting biofilm formation, and 81 genes were associated with defense mechanisms. CAZy analysis revealed a high proportion of glycoside hydrolases (GHs, 35.43%) and glycosyltransferases (GTs, 29.71%).

#### 3.3.2. Virulence

No virulence factors were detected.

#### 3.3.3. Mining of Secondary Metabolite Gene Clusters in MG0718

**Figure 9:**
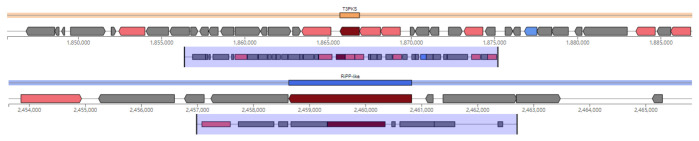
Prediction of secondary metabolites of MG0718 using the AntiSMASH database.

In this study, antiSMASH 5.0 was employed to mine the genome of strain MG0718 for secondary metabolite biosynthetic gene clusters (BGCs). As shown in Figure 9, two putative BGCs were identified. The first was annotated as a Type III polyketide synthase (T3PKS) cluster, harboring the core gene mvaS, which encodes 3-hydroxy-3-methylglutaryl-coenzyme A (HMG-CoA) synthase. The second cluster was predicted as a ribosomally synthesized and post-translationally modified peptide (RiPP)-like cluster, carrying the core gene lagD. This gene encodes an ATP-binding cassette (ABC) transporter accessory protein responsible for the processing and secretion of bacteriocins (e.g., lactococcin G), constituting the key genetic basis for the strain’s antimicrobial peptide production.

#### 3.3.4. Antimicrobial Activity and Bacteriocin Production

**Figure 10:**
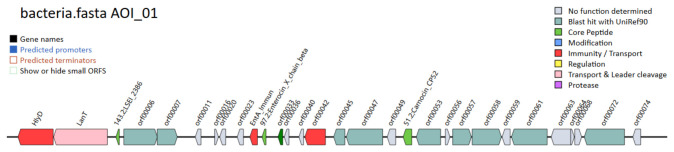
Major bacteriocin protein-encoding gene clusters searched through the BAGEL4 bacteriocin database.

The bacteriocin detection tool BAGELv.4.0 identified a putative bacteriocin gene cluster within the MG0718 genome (Figure 10), in which all four core proteins were classified as Class IIb bacteriocins. Their sequence identities were determined to be 43.182% (E-value = 6.89 × 10^−9^), 46.154% (E-value = 1.07 × 10^−13^), and 32.727% (E-value = 5.03 × 10^−21^), respectively. Notably, one of the bacteriocin gene clusters exhibited no matching reference cluster, indicating the potential presence of a novel Class IIb bacteriocin, which warrants further investigation.

#### 3.3.5. Annotation of the Extracellular Polysaccharide Gene Cluster of MG0718

**Figure 11:**
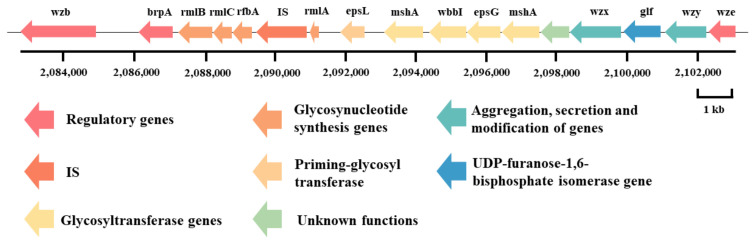
MG0718 EPS synthesis gene cluster.

In LAB, genes responsible for EPS synthesis often exist as single genes or gene clusters; this structure confers efficiency and precision to the EPS synthesis pathway. Currently, genes involved in LAB EPS biosynthesis generally include those encoding nucleoside synthases, polymerases, invertases, and the CpsB/CpsD proteins involved in the regulation of EPS biosynthesis. Based on gene annotation and BLAST alignment, the gene clusters and sequence information of MG0718 are shown in Figure 11. The chromosome of MG0718 contains a single EPS gene cluster with a length of 20,346 bp and a modular structure. The EPS gene cluster is flanked by the regulatory protein genes *wzb*, *brpA*, and *wze.* At the center of the cluster are four glycosylate synthase genes (*rmlB*, *rmlC*, *rfbA*, and *rmlA*), one initiator glycosyltransferase gene (*epsL*), three glycosyltransferase genes (*mshA*, *wbbI*, and *epsG*), two genes encoding transmembrane proteins (*wzx* and *wzy*), one gene encoding UDP-furan galactose isomerase (*glf*), one gene encoding a transposase (IS element), and one domain protein of unknown function. Based on the above, the gene cluster of MG0718 EPS is consistent with known gene cluster structures and contains genes related to EPS synthesis.

#### 3.3.6. Antibacterial Gene

As shown in Table 5, functional screening was performed on 15 genes of MG0718, resulting in the identification of 5 genes encoding bacteriocins and 7 genes encoding lysozymes. Therefore, it is speculated that MG0718 possesses specific antibacterial efficacy at the genetic level.

#### 3.3.7. Antistress Gene Analysis of MG0718

A comprehensive analysis of the whole genome of strain MG0718 identified a total of 198 genes associated with stress resistance (Table 6). Among these, genes encoding antioxidant activity were the most prevalent, numbering 45, followed by 24 genes encoding proteins related to osmotic balance.

## 4. Discussion

A key characteristic of potential probiotic strains, particularly within the field of food microbiology, is their antimicrobial activity [30]. This property is crucial as it enables these strains to inhibit the growth of pathogenic and spoilage microorganisms, thereby enhancing food safety and quality [31]. During growth and metabolism, lactic acid bacteria (LAB) produce and secrete a variety of bioactive metabolites—including short-chain fatty acids, organic acids, glyoxylic acid, peptides, hydrogen peroxide, exopolysaccharides, and bacteriocins [32]—which mediate cell signaling and confer probiotic effects upon the host [33]. In the present study, MG0718 exhibited antimicrobial activity against both Gram-positive and Gram-negative bacteria in vitro. Furthermore, mice pre-treated with MG0718 achieved a 100% survival rate following infection with *S. *Typhimurium. Weight loss induced by *S. *Typhimurium infection was significantly alleviated, and liver damage was mitigated; concurrently, tissue bacterial load decreased, and the inflammatory response was reduced. These findings suggest that MG0718 possesses potent in vivo antimicrobial activity, consistent with the demonstrated anti-infective capabilities of numerous LAB strains [23,34,35]. This broad-spectrum activity aligns with the findings of other researchers [36,37]. Although LAB typically demonstrate higher activity against Gram-positive pathogens [38,39], some studies suggest there is no correlation between the antagonistic activity of lactobacilli and the Gram status of the pathogen [40]. Although we demonstrated MG0718’s protective efficacy against *S*. Typhimurium infection in vivo, its effect on the overall intestinal microbiome was not assessed. Current evidence suggests that *Lbs. rhamnosus* strains generally exert minimal effects on the global gut microbial community, although specific taxa may be affected [41,42]. Whether MG0718 modulates particular taxa (e.g., enriching beneficial genera or suppressing pathogens) requires direct evaluation through 16S rRNA or metagenomic sequencing in future studies.

In this study, antiSMASH 5.0 was used to mine the genome of strain MG0718 for biosynthetic gene clusters. Two putative clusters were identified: a T3PKS cluster (harboring the core gene mvaS) and a RiPP-like cluster (harboring the core gene lagD). The mvaS gene encodes HMG-CoA synthase, a key enzyme in the mevalonate pathway that supplies C5 precursors for polyketide and terpenoid biosynthesis [43]. This metabolic feature may enable the strain to better regulate carbon flux and resist oxidative stress during fermentation. T3PKS clusters are widely distributed among lactobacilli, suggesting that polyketides produced by this cluster may serve as important metabolites for niche adaptation and physiological resilience [43,44].

The lagD gene in the RiPP cluster encodes a protein responsible for bacteriocin processing and transport. This genetic architecture is typically co-localized with immunity genes, allowing the strain to secrete antimicrobial peptides without self-harm [45,46]. This mechanism likely provides MG0718 with a competitive advantage in complex microbial communities and supports its potential use as a biopreservative strain.

Additionally, BAGEL analysis detected a class IIb bacteriocin cluster with low sequence homology to known references, suggesting that this strain may harbor novel bacteriocins. Functional bacterial gene clusters responsible for producing extracellular cationic peptides (bacteriocins) are typically organized as operons, containing genes involved in bacteriocin synthesis, immunity, ABC transport, and accessory proteins [47]. Therefore, AOI_01 is likely involved in bacteriocin biosynthesis and contributes to the antimicrobial activity of MG0718. However, further investigation revealed that neutralizing the supernatant pH to 7.0 completely eliminated its antimicrobial activity, indicating that organic acids are the primary mediators of the inhibitory effect exerted by MG0718. Consistent with previous findings [48,49], hydrogen peroxide was not the source of antimicrobial activity in MG0718, a result that aligns with the findings of Reuben et al. [50]. Phenotypic studies indicate that organic acids are the primary contributors to the antimicrobial activity of LAB. Apart from using BAGEL to detect bacteriocin clusters, we also conducted a functional screen across the entire genome. Five bacteriocin-encoding genes and seven lysozyme-encoding genes were found. We hypothesize that these genes form part of the genetic foundation for the antimicrobial effects of MG0718. However, machine learning models predicted the presence of Class II bacteriocins and lysozymes in this strain. Secondary metabolite gene clusters in lactic acid bacteria are highly diverse and strain-specific, and many remain uncharacterized. Even when bacteriocin gene clusters are predicted, their expression may not be detectable due to strain specificity or experimental conditions. Therefore, integrating metabolomic and transcriptomic analyses with machine learning models is crucial for guiding the discovery of functional bacteriocins [44].

In addition to bacteriocin clusters, the MG0718 genome also contains an EPS gene cluster. Exopolysaccharides can enhance cell surface hydrophobicity and adhesion. They also regulate the intestinal microenvironment and inhibit pathogen adhesion. In addition, their spatial structures are closely related to antioxidant and immunomodulatory activities [51]. The MG0718 genome contains one EPS gene cluster. It is 20,346 bp long and encodes 17 open reading frames. This cluster provides a complete pathway for polysaccharide synthesis. It includes three regulatory genes (*wzb*, *brpA*, *wze*) and four sugar nucleotide synthesis genes (*rmlB*, *rmlC*, *rfbA*, *rmlA*). These genes control EPS synthesis and supply dTDP-rhamnose precursors. This structure is similar to the EPS clusters of other *Lbs. rhamnosus* strains. Such clusters usually span 18–20 kb and encode 16–17 ORFs [52,53]. However, MG0718 contains only three glycosyltransferase genes (*mshA*, *wbbI*, *epsG*). This number is lower than that of *Lbs. rhamnosus* GG (six) [53] and *Lbs. rhamnosus* ATCC 9595 (five) [52]. Nevertheless, MG0718 retains the minimum functional unit needed for heteropolysaccharide synthesis. Notably, an IS element is inserted upstream of the *rmlA* gene in MG0718. Such insertions are common in *Lbs. rhamnosus* EPS clusters. They often cause genomic plasticity and EPS diversity among strains [54]. In *Lbs. rhamnosus* GG, an IS element also interrupts *rmlA*. However, *Lbs. rhamnosus* GG carries a complete *rmlABCD* operon at another locus as compensation [53]. Whether MG0718 has a similar compensatory mechanism remains to be verified.

Advances in research on LAB have expanded their applications in food fermentation, industrial biotechnology, and medicine, while highlighting the importance of understanding their stress response mechanisms. During production and storage, LAB encounter various stress conditions, including deviations from optimal temperature, osmolarity, and pH, as well as exposure to oxidizing agents [55]. Furthermore, when used as probiotics, LAB must be able to tolerate gastrointestinal stress and resist resident gut microbiota [56,57]. Survival in natural environments requires robust stress adaptation mechanisms. LAB possess a variety of molecular strategies to cope with these stresses both during processing and after ingestion [58].

The small intestine contains 0.03–0.3% bile salts, which can inhibit microbial growth and metabolism. Therefore, bile salt tolerance is a key factor for the survival of LAB in the intestinal environment [59]. In our phenotypic assays, strain MG0718 exhibited tolerance to 0.3% bile salts, although tolerance decreased as the concentration increased. Bile salt tolerance in the genus lactobacilli is primarily mediated by bile salt hydrolases [60] and bile salt transporters [61]. While genes related to bile salt hydrolase were not detected in strain MG0718, a rich set of transporter genes was identified. These abundant transporter genes may facilitate the expulsion of bile salts via bile efflux systems, thereby alleviating osmotic stress and enabling the strain to tolerate low concentrations of bile salts. Tolerance to gastrointestinal fluids is another critical characteristic of probiotics. MG0718 demonstrated robust tolerance in simulated gastric and intestinal fluids, with a substantial population surviving after 2 h of exposure. Acid tolerance tests showed that the strain could maintain viability in a solution at pH 2.5 for 6 h. Its acid tolerance is consistent with the presence of acid-resistance-related genes in its genome, including genes encoding dTDP-glucose 4,6-dehydratase [62], 4-hydroxy-tetrahydrodipicolinate synthase, ATP-dependent Clp endopeptidase proteolytic subunit ClpP [63], and F0F1-type ATPase subunits [64]. These genes likely play a pivotal role in adaptation to low pH environments. In phenotypic assays, strain MG0718 exhibited robust tolerance to both low and high temperatures. After treatment at temperatures ranging from −20 °C to 10 °C for 48 h, its survival rate remained stable, demonstrating remarkable cryotolerance. Conversely, heat treatment reduced its survival rate: viable counts were 6 log10 CFU/mL at 60 °C, and viable bacteria persisted even after treatment at 70 °C.

Compared to other well-studied strains, MG0718 demonstrated superior temperature tolerance. For instance, the widely recognized probiotic strain *Lbs. rhamnosus* GG showed significantly reduced viability at 60 °C and completely lost viability at 70 °C [65]. Similarly, *Lbs. rhamnosus* OF44 exhibited lower activity at 60 °C, indicating weaker heat tolerance [66]. This temperature tolerance is likely attributed to genes regulating thermal adaptation in strain MG0718. Cold shock DNA-binding domain proteins (CSD) and cold shock proteins maintain genome stability and efficient protein synthesis under low-temperature conditions by binding to nucleic acids and preventing the formation of secondary RNA structures [67]. Under heat stress conditions, small heat shock proteins (sHsps) (such as HslO) and transcriptional regulators (such as HrcA) are induced, thereby protecting proteins from heat damage and regulating the expression of stress response genes in MG0718 [68]. Collectively, these mechanisms enhance the strain’s tolerance to dual cold and heat stresses. These traits provide MG0718 with a significant advantage in food and probiotic applications, particularly in environments requiring tolerance to extreme temperatures. The ability of potential probiotic strains to adhere to intestinal cells is a fundamental criterion for probiotic screening [69]. It has been suggested that auto-aggregation—which supports bacterial adhesion to host gastrointestinal epithelial cells and prevents pathogen colonization—should exceed 40% in potential probiotic strains [69,70].

Through whole-genome analysis, this study revealed that the adhesion capability of strain MG0718 relies on various surface proteins capable of binding to host cell receptors or extracellular matrix components. Phenotypic experiments indicated that the auto-aggregation rate of MG0718 reached 87%, which is significantly higher than other common LAB such as *L. acidophilus* (21.4%), *Lbs. rhamnosus* GG (13.1%), and *Bifidobacterium* species, which are typically below 20% [71], This suggests a potential advantage for MG0718 in gastrointestinal colonization. However, its cell surface hydrophobicity was relatively low (16.7% when mixed with xylene). This is consistent with previous studies indicating that many lactobacilli strains, including *Lbs. rhamnosus*, typically exhibit low to moderate hydrophobicity [72]. This low hydrophobicity may be attributed to the presence of surface proteins and exopolysaccharides (EPS), which mask hydrophobic sites on the cell surface [72]. Despite this, the high auto-aggregation capability of MG0718 and the presence of specific surface proteins may compensate for its lower hydrophobicity, thereby enabling effective adhesion to intestinal cells [72]. Notably, low hydrophobicity may also affect the intestinal residence time of MG0718. Previous studies have shown that probiotic colonization is generally transient. In murine models, *Lbs. rhamnosus* GG was eliminated from feces within approximately 7 days after a single gavage, and only in vivo evolution extended its persistence to 14–21 days [73]. Thus, daily administration is likely required to maintain effective intestinal concentrations for wild-type strains. The exact dosing frequency and residence time of MG0718, however, remain to be determined in future studies.

Lactic acid bacteria are widely used in the food and health sectors, playing vital roles in maintaining cell viability, enhancing host health, and adapting to complex environments. Their antioxidant mechanisms allow them to survive in oxidative stress environments, such as the gut, and protect cells from damage caused by reactive oxygen species (ROS) and reactive nitrogen species (RNS) [74]. In this study, we conducted a comprehensive analysis of the oxidative stress resistance genes of strain MG0718 and evaluated its in vitro antioxidant efficacy. The superoxide anion scavenging ability of the strain was 16.5%, which is relatively low compared to most LAB. However, its DPPH radical scavenging ability reached 56.7%, significantly surpassing that of other strains. Previous studies have shown that the DPPH radical scavenging activity of parsley cheese ranges from 31% to 48% [75]. Afify et al. [76] reported that the DPPH scavenging rate of *L. plantarum* C88 was 53.1%, slightly lower than that of strain MG0718. Ferricyanide reduction experiments showed that the total reducing power of strain MG0718 reached 91.1%, which may be associated with the high number of genes encoding oxidoreductases and antioxidant proteins. These antioxidant genes cover multiple aspects, including sulfur metabolism, oxidoreductases, antioxidant proteins, and transcriptional regulators, collectively constituting a complex defense network. Key components include cysteine desulfurase and thioredoxin involved in sulfur metabolism [77,78]. SDR family oxidoreductases and NADP-dependent oxidoreductases maintain redox balance [79,80], while peroxiredoxins and glutathione peroxidase serve to mitigate oxidative damage [81,82]. Transcriptional regulators (such as the DeoR/GlpR and Crp/Fnr families) regulate the expression of antioxidant genes according to the cellular redox state [83,84,85]. The synergistic action of these genes enhances the strain’s tolerance to oxidative stress, ensuring its survival in complex environments. Future research could further investigate the expression patterns and regulatory mechanisms of these stress-resistance genes under different environmental conditions, as well as their interactions. This will deepen our understanding of the stress resistance of strain MG0718 and provide a solid theoretical foundation for its application in food and pharmaceutical fields.

The genomic and phenotypic traits of MG0718 reflect strong niche adaptation to its isolation source. This strain was isolated from traditional sour bamboo shoots in Guangxi, a naturally fermented vegetable product. Unlike intestinal strains, MG0718 evolved under open, highly acidic, and microbially competitive fermentation conditions. This ecological pressure likely selected for its unique genotype. Phenotypically, its robust acid tolerance, broad-spectrum antibacterial activity, and thermal stability meet the functional demands of fermentation survival and food processing. These findings emphasize that probiotic characteristics are profoundly habitat-imprinted. Therefore, strain-specific evaluation must consider the original ecological niche rather than extrapolating from human-derived model strains.

## 5. Conclusions

MG0718 exhibited broad-spectrum inhibitory effects against pathogenic bacteria such as *E. coli* *in vitro* (inhibition zone: 13.67 ± 1.56 mm). Prophylactic administration for 7 days achieved 100% survival in mice infected with *S.* Typhimurium (vs. 0% in the infection group), significantly reduced tissue bacterial loads, and alleviated infection-induced weight loss and liver damage. The strain possesses superior biological traits, including potent antioxidant capacity (DPPH scavenging: 56.7%; total reducing power: 91.1%), robust gastrointestinal tolerance (only 1.72, 0.78, and 1.11 log10 reductions after exposure to pH 2.5, artificial gastric fluid, and intestinal fluid, respectively), and strong auto-aggregation (>87%). Whole-genome analysis (2,574,565 bp; 2813 CDS) identified 203 stress-related protein genes, one bacteriocin gene cluster, one EPS gene cluster, and two secondary metabolite clusters, providing a genetic basis for its probiotic features. Collectively, these findings designate MG0718 as a highly promising probiotic candidate. Its exceptional adaptability to food processing conditions and the digestive environment, combined with its safety profile, bridges the gap between food microbial resources and human health promotion.

## Figures and Tables

**Table 1:** MG0718 CFS acid drainage results.

Strains	MG0718 CFS		MG0718 CFS-pH 6.5		MRS + Lactic Acid		MRS + Acetic Acid	
	Inhibitory Circle Diameter/mm	Sensitivity	Inhibitory Circle Diameter/mm	Sensitivity	Inhibitory Circle Diameter/mm	Sensitivity	Inhibitory Circle Diameter/mm	Sensitivity
*S.* Typhimurium	11.00 ± 1.53	++++	0	-	0	-	0	-
*S. aureus* ATCC 6583	11.33 ± 1.56	++++	0	-	0	-	0	-
*E. coli* ATCC 25922	13.67 ± 1.56	++++	0	-	0	-	0	-

Interpretation criteria for the deferred agar spot test: Diameter > 10 (++++);  no inhibition (-).

**Table 2:** MG0718 CFS exclusion peroxidase results.

Strains	MG0718 CFS		MG0718 CFS + H_2_O_2_		MRS + H_2_O_2_	
	Inhibitory Circle Diameter/mm	Sensitivity	Inhibitory Circle Diameter/mm	Sensitivity	Inhibitory Circle Diameter/mm	Sensitivity
*S. *Typhimurium	11.00 ± 1.53	++++	0	-	0	-
*S. aureus* ATCC 6583	11.33 ± 1.56	++++	0	-	0	-
*E. coli* ATCC 25922	13.67 ± 1.56	++++	0	-	0	-

Interpretation criteria for the deferred agar spot test: Diameter > 10 (++++); no inhibition (-).

**Table 3:** MG0718 CFS deproteinization results.

Strains	MG0718 CFS		MG0718 CFS + Pepsin		MG0718 CFS + Proteinase K		MG0718 CFS + Trypsin		MG0718 CFS + Chymotrypsin	
	Inhibitory Circle Diameter/mm	Sensitivity	Inhibitory Circle Diameter/mm	Sensitivity	Inhibitory Circle Diameter/mm	Sensitivity	Inhibitory Circle Diameter/mm	Sensitivity	Inhibitory Circle Diameter/mm	Sensitivity
*S. *Typhimurium	11.00 ± 1.53	++++	10.67 ± 0.67	++++	0	-	0	-	0	-
*S. aureus* ATCC 6583	11.33 ± 1.56	++++	8.33 ± 0.67	+++	0	-	0	-	0	-
*E. coli* ATCC 25922	13.67 ± 1.56	++++	9.00 ± 0.67	+++	0	-	0	-	0	-

Interpretation criteria for the deferred agar spot test: Diameter > 10 (++++); Diameter between 6 and 10 mm (+++); no inhibition (-).

**Table 4:** Fundamental characteristics of MG0718.

Category	Peoperty
Protein-coding genes	2813
tRNA genes	59
Total gene length	2,574,565
Average gene length	891
GC content in gene region	47%
Gene density (genes/Mb)	983
Gene/Genome (%)	85%
Intergenic region length	432,580

**Table 5:** The antimicrobial protein encoded by the MG0718 genome.

Kinds	Product	Locus
Tacteriocin	Bacteriocin immunity protein	MG0718_02349, MG0718_02340, MG0718_02329
	Bacteriocin	MG0718_02342
	class IIb bacteriocin, lactobin A/cerein 7B family	MG0718_02341
Lysozyme	N-acetylmuramidase	MG0718_01057
	lysozyme M1 (1,4-beta-N-acetylmuramidase)	MG0718_01972
	LysM peptidoglycan-binding domain-containing protein	MG0718_01365, MG0718_01131, MG0718_01491, MG0718_00480
	lyzozyme	MG0718_01025
Antitoxins	Toxin-Antitoxin System, Antitoxin Component	MG0718_02478
	type II toxin-antitoxin system mRNA interferase toxin, RelE/StbE family	MG0718_00487
	PhoH family protein	MG0718_01532

**Table 6:** MG0718 stress resistance genes.

Pressure	Protein Name and Location (Number)	Total
Cold	cold-shock DNA-binding domain protein, cold-shock protein, cold shock protein CspB	3
Heat	small heat shock protein, Hsp33 family molecular chaperone HslO, Hsp20/alpha crystallin family protein, heat-inducible transcriptional repressor HrcA, CtsR family transcriptional regulator	5
Phage	WYL domain-containing protein, Pilus-specific protein, ancillary protein involved in adhesion	3
Acid	ClC family H(+)/Cl(−) exchange transporter, HAMP domain-containing histidine kinase(9), alkaline shock response membrane anchor protein AmaP(1), F0F1 ATP synthase subunit A, F0F1 ATP synthase subunit delta, ATP synthase F0 subunit B, ATP-dependent Clp endopeptidase proteolytic subunit ClpP(2), 4-hydroxy-tetrahydrodipicolinate synthase, dTDP-glucose 4,6-dehydratase(2)	18
Na^+^/H^+^	2-hydroxycarboxylate transporter family protein, AI-2E family transporter(2), sodium:proton antiporter(2), cation:proton antiporter(2), sodium ion-translocating decarboxylase subunit beta, elongation factor(6)	14
Adhesion	VWA domain-containing protein, LapA family protein, Conserved extracellular matrix binding protein, fibronectin/fibrinogen-binding protein, fibrinogen-binding protein, isopeptide-forming domain-containing fimbrial protein	6
Antioxidant activity	Cysteine desulfurase(3), NADP-dependent oxidoreductase(2), Gfo/Idh/MocA family oxidoreductase(3), SDR family oxidoreductase(7), LPXTG cell wall anchor domain-containing protein(11), NAD(P)H dehydrogenase(2), NADH-dependent flavin oxidoreductase, NAD(P)H-dependent glycerol-3-phosphate dehydrogenase, NADP oxidoreductase(4), iron-sulfur cluster biosynthesis family protein, thioredoxin(3), peptide-methionine (S)-S-oxide reductase(2), rhodanese-like domain-containing protein, NAD(P)/FAD-dependent oxidoreductase(4), NAD(FAD)-dependent dehydrogenase, NAD-dependent deacetylase(2), NAD(P)H-hydrate dehydratase, NAD(P)-dependent oxidoreductase(4), NAD dependent epimerase/dehydratase family protein, peroxiredoxin(2), NAD-dependent epimerase/dehydratase family protein, redox-regulated ATPase YchF, NADH oxidase, DeoR/GlpR transcriptional regulator, Transcriptional regulator, xre family(2), L-lactate dehydrogenase, cytochrome d ubiquinol oxidase subunit II(2), class II fumarate hydratase, Short-chain dehydrogenase/reductase SDR, glutathione peroxidase, Dyp-type peroxidase, flavodoxin(5), dioxygenase, redox-sensing transcriptional repressor Rex(2), YitT family protein(3), peptide-methionine (R)-S-oxide reductase, ferredoxin, thioredoxin-disulfide reductase, Crp/Fnr family transcriptional regulator, transcriptional regulator Spx, NADPH-dependent oxidoreductase, peroxide stress protein YaaA, FAD/NAD(P)-binding protein, fructosamine-3-kinase, thiol reductant ABC exporter subunit CydD, NAD-dependent epimerase/dehydratase family protein	85
Osmotic pressure balance	ABC transporter permease(34), ABC transporter permease/substrate-binding protein, cobalt ABC transporter permease(2), antimicrobial peptide ABC transporter permease component, proline/glycine betaine ABC transporter permease, phosphate ABC transporter permease PstA, bacitracin ABC transporter permease, iron export ABC transporter permease subunit FetB, osmoprotectant ABC transporter substrate-binding protein, cation transporter(6), potassium transporter(2), sodium:solute symporter, Chloride channel protein, N-acetylmuramoyl-L-alanine amidase, large-conductance mechanosensitive channel protein MscL, mechanosensitive ion channel family protein, Gx transporter family protein, Cation-transporting ATPase, 1-acyl-sn-glycerol-3-phosphate acyltransferase(3)	61
universal stress protein (UspA)	universal stress protein(3)	3
Summary		198

## Data Availability

The datasets used and/or analysed during the current study are available in the NCBl Sequence ReacArchive repository [CP196043]. The GenBan accession number for accessing the *Lacticaseibacillus rhamnosus* MG0718 genome sequence is [CP196043].

## Supplementary Materials

The following supporting information can be downloaded at: https://www.mdpi.com/article/10.3390/microorganisms14061290/s1, Figure S1. Antipathogenic activity; a: *E. coli*; b: *S. aureus*; c: *S.* Typhimurium. Figure S2. Characterization of MG0718 antimicrobial substances a: *E. coli*; b: *S. aureus*; c: *S. *Typhimurium; A: Acid excretion test: 1: MG0718 CFS, 2: MG0718 CFS-pH6.5, 3: MRS + lactic acid, 4: MRS + acetic acid; B: Hydrogen peroxide excretion test: 1: MG0718 CFS, 2: MG0718 CFS + H_2_O_2_. 3: MRS + H_2_O_2_; C: Protein removal test: 1: MG0718 CFS, 2: MG0718 CFS + pepsin, 3: MG0718 CFS + proteinase K, 4: MG0718 CFS + trypsin, 5: MG0718 CFS + chymotrypsin.

## Abbreviations

bp	base pairs	BS	bacterial suspension
CDSs	protein-coding genes	CSD	Cold stress DNA-binding domain proteins
CFS	Cell-free supernatant	DPPH	1,1-Diphenyl-2-picrylhydrazyl
CAZy	Carbohydrate-Active enZymes	GIT	gastrointestinal tract
GO	Gene Ontology	GHs	glycoside hydrolases
COG	Clusters of Orthologous Groups of proteins	GTs	glycosyltransferases
KEGG	Kyoto Encyclopedia of Genes and Genomes	Hsp	Heat shock proteins
*Lbs. rhamnosus*	*Lacticaseibacillus rhamnosus*	LAB	lactic acid bacteria
MRS	de Man, Rogosa and Sharpe	LB	Luria–Bertani
PBS	phosphate-buffered saline	NS	Normal Saline Solution
SM	secondary metabolite	PLs	polysaccharide lyases
ROS	Reactive Oxygen Species	O_2_^−^	Superoxide Anion Radical
T3PKS	type III polyketide synthase	RNS	Reactive Nitrogen Species
WGS	Whole Genome Shotgun	TP	Total Reducing Power
uspA	universal stress protein		
