# Supplementary material for: Integrative Phenotypic and Genomic Analysis Reveals Antimicrobial and Stress-Resistance Mechanisms of Lacticaseibacillus rhamnosus MG0718 as a Promising Probiotic Candidate for Food Applications
Source: Microorganisms. 2026 Jun 7;14(6):1290. doi: 10.3390/microorganisms14061290 (PMC13302913; doi:10.3390/microorganisms14061290)
Supplement: Supplementary file 1 [file microorganisms-14-01290-s001.zip › microorganisms-4319628-supplementary.pdf]

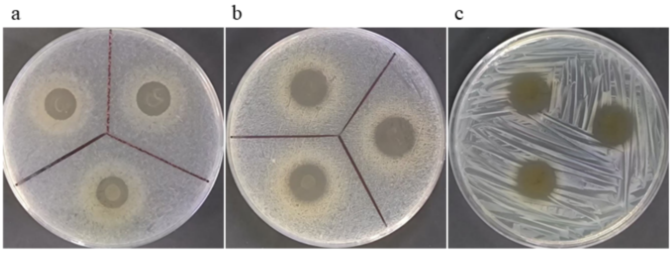


**Figure S1.** Antipathogenic activity; a: *E. coli*; b: *S. aureus*; c: *S.* typhimurium.

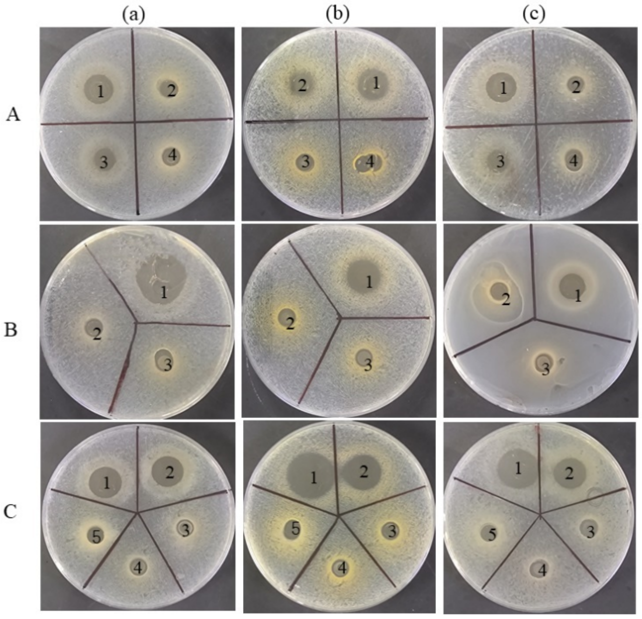


**Figure S2.** Characterization of MG0718 antimicrobial substances a: *E. coli*; b: *S. aureus*; c: *S.* typhimurium; A: Acid excretion test: 1: MG0718 CFS, 2: MG0718 CFS-pH6.5, 3: MRS + lactic acid, 4: MRS + acetic acid; B: Hydrogen peroxide excretion test: 1: MG0718 CFS, 2: MG0718 CFS + $H_2O_2$. 3: MRS + $H_2O_2$; C: Protein removal test: 1: MG0718 CFS, 2: MG0718 CFS + pepsin, 3: MG0718 CFS + proteinase K, 4: MG0718 CFS + trypsin, 5: MG0718 CFS + chymotrypsin.
